# The Relationship Between Sexual Compulsivity, Emotional and Spiritual Distress of Religious and Non-religious Internet Pornography Users

**DOI:** 10.1007/s10943-020-01152-y

**Published:** 2021-02-13

**Authors:** Jason T. Hotchkiss

**Affiliations:** Cornerstone University, 1001 E Beltline Ave NE, Grand Rapids, MI 49525 USA

**Keywords:** Religiosity, Religion, Internet pornography, Emotional distress, Spiritual distress, Sexual compulsivity

## Abstract

This study sought to examine the relationship between the sexual compulsivity, emotional and spiritual distress of religious and non-religious adults who sought assessment for pornography addiction on the Internet. Religious (*n* = 350) and non-religious (*n* = 114) data were analyzed separately with a one-way between-subjects multivariate analysis of variance. The Kalichman Sexual Compulsivity Scale was used to divide the religious and non-religious into three groups: non-sexually compulsive (NCs); moderately sexually compulsive and sexually compulsive (SCs). All of the dependent variables, except age, were significantly higher for SCs than NCs for the religious. For the non-religious, all of the dependent variables, except age and time spent viewing Internet pornography (IP), were significantly higher for SCs than NCs. The non-religious spent significantly more time viewing IP than the religious. Yet, the religious were significantly more sexually compulsive. Emotional distress and spiritual distress were found to be significantly higher for SCs than the NCs regardless of religiosity. The non-religious were significantly more anxious and stressed than the religious. Specific religious affiliations did not have any significant bearing on the degree of sexual compulsivity. Religious practice, being associated with less viewing of IP, suggests the likelihood that moral reasons may provide some rationale for not viewing IP. At the same time, religious practice might reinforce shame in the addiction cycle thus religious individuals may be more at-risk to developing a compulsive pattern of viewing IP. The implications of the findings and suggestions for future research are presented.

## Introduction

The Internet is a communication and information network of unprecedented impact and influence. “There is no medium more globally accessible than the Internet” (Cooper et al. 2004). The Internet is the global market for information, goods and social connections. With this global accessibility comes risk. Over 50% of all Internet traffic is associated with sex-related material (McNair 2002). “The tremendous communication potential of the Internet, coupled with the allure of sexual media, has led to a renewed interest in the social effects of pornography” (Wright 2012, p. 67).

### Problematic Use

There is a debilitating side to the Internet. Exposure to and repeated use of Internet pornography (IP) is known to cause psychosocial-spiritual distress and is associated with negative affect states (Carnes 2001). Problematic IP use has been positively correlated with loneliness (Yoder et al. 2005); loss of control (Carnes 2001); jeopardizing aspects of life (Cooper et al. 2000); family conflicts (Manning 2006); and legal and financial consequences (Delmonico and Miller 2003). Too much IP use may be way to self-medicate underlying issues such as depression, anxiety and obsessive–compulsive disorders (Young 2007).

To understand those individuals for whom IP use has become problematic, the degree of compulsivity must be considered. Compulsive sexual behavior impacts between 3 and 6% of the US population (Kuzma and Black 2008). Although this estimate does not specifically report on those with compulsive IP use (CIPU), 53% of individuals reporting compulsive sexual behavior also indicated that IP use was a distressing aspect of their behavior. From this, an estimated 1.5% to 3% of the US adult population is beset by CIPU (Kuzma and Black 2008). Other studies show that these figures on prevalence may be low. For example, a study of over 9000 Internet users found that between 9 and 15% of the participants self-reported that their use of the Internet for sexual purposes caused them distress (Cooper et al. 2004).

### Sexual Compulsivity

The nature of sexual compulsivity must be operationalized for valid assessment. Coleman (1986), a pioneer in studying problematic sexual behavior, advised using the term “sexual compulsivity” to describe a substantially broader range of both paraphilic and nonparaphilic sexual behavior disorders. The term “sexual compulsivity” has been adopted by other clinical investigators (Bancroft and Vukadinovic 2004; Cooper et al. 2000, 2001; Kuzma and Black 2008). Sexual compulsivity has been characterized by the insistent, repetitive and intrusive urges to engage in sexual behaviors (Kalichman and Rompa 1995). Because the term compulsivity can imply specific pathology and can be confused with the *DSM* term obsessive–compulsive (APA 2013), the present study used the term compulsive IP use to encompass all such use that impacts a person’s general social, occupational and interpersonal areas of functioning and avoid the potentially pathologizing term “addiction.”

Cooper et al. (2000) were one of the first groups of researchers to empirically examine the attributes and consumption patterns of individuals who use the Internet for sexual purposes. The SCS was the primary assessment instrument, which divided the participants (*n* = 9265) into four groups: non-sexually compulsive (*n* = 7738), moderately sexually compulsive (*n* = 1007), sexually compulsive, (*n* = 424) and cybersex compulsive (*n* = 96; Cooper et al. 2000).

### Time Spent Viewing IP

Sexual compulsivity is linked to time spent online for sexual purposes. Researchers investigating the addictive potential of the Internet regarding both sexual and non-sexual use have noted correlations between time spent online and negative consequences reported by users (Cooper et al. 2000). There is much discussion concerning general Internet and Internet pornographic usage and the range by which it is measured as normative to excessive usage (Cooper et al. 2000; Wetterneck et al. 2012).

Kubey et al. (2001) reported that average Internet usage (encompassing all aspects of the Internet, including IP use) ranged from “2.4 to 3.2 h per week” (p. 366). However, “pathological users averaged 8.5 h per week and reported significant loneliness on the UCLA loneliness scale” (Kubey et al. 2001, p. 366).

Another study found that the time of online sexual activity was correlated with the distressing consequences of money spent and illegal sexual material assessed online. Delmonico and Miller (2003) found that sexually compulsive males and females (*n* = 6088, males = 82%, females = 18%) averaged approximately 10 to 11 h each week in online sexual behavior within their 20 to 22 h per week of total Internet use. “The ratio of total online time to time spent online in pursuit of sexual behaviors was found to be meaningful, since sexual compulsives spent significantly more time engaged in online sexual behavior than non-sexual compulsives” (p. 261).

### Escaping and Assuaging Negative Emotions

When an individual spent excessive time viewing IP, despite negative consequences and avoided responsibilities, they are attempting to escape and/or assuage negative emotions such as depression, anxiety or stress (Cooper et al. 2000, 2001). Wright (2012) argued “that negative affective states make the application of risky sexual scripts (i.e., casual, unprotected sexual behavior) acquired or activated by sexual media more likely” (p. 69). Those who use IP compulsively and who are unhappy with their lives may not fully understand “the potential long-term consequences of their behavior, or are too emotionally depleted to exercise sexual self-restraint” (Wright 2012, p. 69). In this sense, compulsive use of pornography may be a means of self-medicating depression or anxiety.

Cooper (1998) initial study and subsequent research suggest the importance of internal psychological factors and the user's motivation on the nature and extent of harm consequences (Cooper et al. 2000, 2001). In support of the centrality of mood alteration, Cooper et al. (2001) revealed three subtypes of motivation for at-risk users: (a) the stress reactive user, who uses IP to decrease anxiety and escape during times of stress; (b) the depressive user, who uses online sexual activity to find relief from depressed or dysphoric symptoms; and (3) the fantasy user, who uses the Internet to engage in sexual fantasies that he or she may not act out in real life. Thus, CIPU affects a person’s emotional distress. Either an individual’s dependence on CIPU lowers well-being or individuals with diminished emotional distress resort to CIPU to assuage negative feeling states (Kuzma and Black 2008).

### Positive Aspects

Apart from the potential negative impacts, non-compulsive IP use may also have some healthy benefits. Some studies have shown that pornography might have no effect or even possess beneficial effects (Bridges et al. 2003; Davies 1997; Hald and Malamuth 2008). For gay individuals, the Internet is a useful source for building virtual communities in regard to sexual interests (Cooper et al. 2000). As a shared activity in intimate relationships, IP allows for more candid dialog, sexual experimentation (Albright 2008; Hald and Malamuth 2008), sexual intimacy education (Daneback et al. 2006) and sexual stimulation (Philaretou et al. 2005).

### Religion, Spirituality and Compulsivity IP Use

The phenomenon of CIPU and how it relates to religiosity has been studied in a limited way. Most studies have been entirely focused on Christians, sampled only college students and/or made no comparison to non-religious individuals (Abell et al. 2006; Baltazar et al. 2010; Nelson et al. 2010; Short et al. 2015). The reason that Christian populations have been studied heavily is because many Christians (and other religious people) regard IP as sinful and to be avoided. Even though, these studies largely Christian populations, they do reveal some aspects of the relationship between religious and IP use.

Nelson et al. (2010) found that individuals who do not use pornography have higher levels of recent and past religious practices and past family religious practices lower levels of depression, than those who used pornography. Some research shows that religiosity is negatively correlated with pornography use (Baltazar et al. 2010; Nelson et al. 2010; Short et al. 2015). However, each of these studies researched general IP use and did not evaluate the degree of sexual compulsivity of the IP use. Religiosity was not a protective factor against CIPU for Christians (Abell et al. 2006).

O'Reilly et al. (2007) surveyed 305 college students (59% male), of whom 70% considered themselves to be religious. They found that over 40% of individuals polled admitted viewing pornography one to two times a week and over 92% reported that they had viewed pornography at least once in their lifetime. Again, religiosity did not appear to be a protective factor against IP use for the religious.

A Christianity Today International (CTI) study reported that clergy members, pastors and other leaders have the same rate of IP use as those who are non-religious. The study conducted by the editors of (CTI) queried 549 church leaders around the USA, including pastors and volunteer leaders, regarding their Internet use. A total of 84% of the respondents identified themselves as a pastor of varying capacity. A total of 37% of church leaders reported that IP use was a current struggle. (Galli 2001). Lee (2009) conducted a follow-up survey to the CTI study in order to determine current consumption rates of online sexually explicit content. He queried 134 ministers of a nationwide Protestant congregation and found that the percentage of church leaders reporting a current struggle with IP was nearly the same as the original CTI study (Galli 2001).

The three factors of accessibility, affordability and anonymity reported by Cooper et al. (2000) may provide some explanation of why the protectiveness of certain values and practices such as religion may be neutralized by the uniqueness of cyberporn use. Moreover, Abell et al. (2006) argued that the use of pornography may be deemed as a less “sinful” outlet for sexual appetite than sexual desires actualized in real non-marital relationships.

Religion can either create positive or negative dynamics concerning mental health issues (Abraham 1994; Birchard 2004; Templin and Martin 1999). Abraham (1994) contends that the aspect of religiosity can not only have the capability to enhance mental health for the individual as related to sexuality; but likewise, can also disconnect and isolate the person from her/his sexuality, which can lead to various problems. The dynamics such as guilt and shame integrate into one’s religious beliefs and can also influence sexual behaviors in a detrimental manner.

Birchard (2004) discusses how for the religious individual there may be compulsive cycles in which there are control and release aspects in sexual behavior, which can exacerbate these problems. He argues that sexual addiction and religious behavior are interlinked in that the sexual behavior represents the release part of the cycle and the religious behavior represents the control part of the cycle. “The characteristics of the religious behavior (i.e., repentance, confession, diligence, service) actually move the client to a sense of neediness or entitlement and thus back into the sexually addictive behavior. Unless this cycle is understood and aborted these apparently antithetical behaviors contribute to one another” (p. 86).

In summary, studies have consistently and alarmingly shown that religion does not appear to offer any kind of a protective or buffering effect against cyberporn. Certainly, the relative ease with which cyberporn can be accessed may be especially contributory to the ineffectiveness of religious buffering. Cooper et al. (2002) have provided some explanation of why the protectiveness of certain values and practices such as religion may be somewhat neutralized by the uniqueness of IP.

How religious and non-religious adults compare in their degree of CIPU and the associated emotional/spiritual distress has not yet been studied.

### Purpose

This study sought to examine the relationship between sexual compulsivity and emotional/spiritual distress of religious and non-religious adults who sought assessment for pornography addiction on the Internet. The participants in this study expressed concerns regarding their IP use and sexual addiction/compulsivity.

## Method

The Sexual Compulsivity Scale (SCS) assessed the degree of compulsive IP use. Participants were asked about the hours spent online viewing IP per week. Emotional distress was assessed by the depression anxiety stress scale-21 (DASS-21). Religious and spiritual distress was assessed by the Religious and Spiritual Struggles Scale (RSSS). Demographic questions were asked regarding: age, gender, education, religious preference, religious affiliation and religious attendance.

### Measures

#### Depression Anxiety Stress Scale-21 (DASS-21)

The DASS-21 is a public domain instrument that uses 21 questions to assess three negative affect states with three subscales: depression, anxiety and stress (Lovibond and Lovibond 1995a, 1995b). The DASS-21 is an instrument with excellent reliability and validity. Henry and Crawford (2005) found the DASS-21 reliable and internally consistent.

#### Religious and Spiritual Struggles Scale (RSSS)

This measure consists of 26 items that assess Spiritual struggle and adjustment experiences of six different types of spiritual struggle across the three domains noted earlier: Intrapersonal (morality/guilt, doubt and existential concerns about ultimate meaning), Interpersonal and Supernatural (divine, demonic) (Exline et al. 2014). Participants rated the extent to which they have had the experiences described by each of the items on a five-point scale 1 (not at all) to 5 (a great deal). Data from a large university sample supported the predictive, convergent and discriminant validity of this measure and good internal consistency in this sample; Cronbach’s α was 0.91 (Exline et al. 2014).

#### Sexual Compulsivity Scale (SCS)

SCS consists of 10 items, 4-point Likert-type measure designed to assess sexual compulsivity (Kalichman and Rompa 1995). Seven of the items encompass the construct of hypersexuality and address how an individual reacts to sexual desires. Three of the items focus more on the construct of sexual preoccupation—the negative effects of sexual thoughts and behaviors. The SCS has been shown to predict problematic sexual behaviors and it predicts internal events conducive to engaging in sexual behaviors that are likely to be problematic. The SCS has good validity, reliability and internal consistency (*α* = 0.86) and has test–retest reliability of 0.64 (Kalichman and Rompa 1995). A cutoff score of 24 to below 30 has been used to indicate moderate sexual compulsivity, a score 30 or above indicates high sexual compulsivity (Cooper et al. 2000; Parsons et al. 2001; Reid 2007).

SCS scores below 24 operationally defined non-sexually compulsive users, SCS scores of 24–30 defined moderately sexually compulsive users, scores of 30 or above defined sexually compulsive users.

### Procedures

Participants visiting the web site (http://www.growthclimate.com) voluntarily completed the online form entitled *Assessing Pornography Addiction* (Skinner 2015) that delivered the demographic questionnaire, SCS, DASS-21, RSSS and time reviewing porn. After completing the measures, the responses were submitted with no Internet or personal identifiers provided to the authors.

Data collection over the period of approximately 3.5 years resulted in a total of 964 participants. To preserve data quality, 350 incomplete and duplicate (from the same username or Internet Protocol address) surveys were removed, leaving 614 completed surveys. Only respondents (*N* = 464, 75.5%) who reported concerns regarding their IP use were included in the present study.

### Demographics

As reported in Table 1, participants were 95.4% male (*n* = 443) and 4.6% female (*n* = 21). The mean age for the participants was 33.4 (SD = 16.4, range = 18 to 69 years of age). There were 350 religious and 114 non-religious participants.

**Table 1:** Demographic data of participants

*n* (% of sample)	
*Gender*	
Male	443 (95.5)
Female	21 (4.5)
*Age* (*M* = 33.4, SD = 16.4)	
18–24	94 (20.3)
25–29	104 (22.4)
30–39	141 (30.4)
40–44	81 (17.5)
50–54	35 (7.5)
60–69	9 (1.9)
*Religiosity*	
Religious	350 (75.4)
Non-Religious	114 (24.6)
*Relationship status*	
Never married	64 (13.8)
Cohabiting	75 (16.2)
Separated	19 (4.1)
Divorced	5 (1.1)
Married	301 (64.9)
*Ethnicity*	
Non-Hispanic White	361 (77.8)
Hispanic	22 (4.7)
Black	28 (6.0)
Asian	32 (6.9)
Other	21 (4.5)

The ethnicity of participants was the following: 77.8% identified as non-Hispanic White, 4.7% as Hispanic American, 6.0% African American, 6.9% Asian American and 4.5% other (see Table 1).

### Analysis

Using the Kalichman SCS operational definitions: the religious broke down into the following SCS categories, as presented in Table 2: non-sexually compulsive (*n* = 177); moderately sexually compulsive (*n* = 100); and sexually compulsive (*n* = 73). The non-religious included: non-sexually compulsive (*n* = 68); moderately sexually compulsive (*n* = 32); and sexually compulsive (*n* = 14).

**Table 2:** Mean and standard deviation for time spent viewing porn, DASS21 Scale Scores, Religious and Spiritual Struggle Scale Score by sexual compulsivity categories for the religious and non-religious

Sexual compulsivity scale categories	Mean	SD	*N*
*Hours viewing Internet pornography per week*			
Non-compulsive			
Yes	6.59	7.96	177
No	10.42	10.94	68
Total	7.66	9.03	245
Moderately compulsive			
Yes	8.98	8.19	100
No	12.89	7.41	32
Total	9.92	8.15	132
Sexually compulsive			
Yes	12.96	14.59	73
No	16.49	20.62	14
Total	13.53	15.63	87
Total			
Yes	8.60	10.05	350
No	11.86	11.79	114
Total	9.40	10.59	464
*DASS21 Depression Scale*			
Non-compulsive			
Yes	4.79	4.48	177
No	5.44	5.13	68
Total	4.97	4.67	245
Moderately compulsive			
Yes	7.69	4.69	100
No	10.72	5.14	32
Total	8.42	4.95	132
Sexually compulsive			
Yes	12.34	5.05	73
No	12.14	6.80	14
Total	12.31	5.32	87
Total			
Yes	7.19	5.49	350
No	7.75	6.02	114
Total	7.33	5.62	464
*DASS21 Anxiety Scale*			
Non-compulsive			
Yes	2.29	2.75	177
No	1.99	2.32	68
Total	2.21	2.64	245
Moderately compulsive			
Yes	4.06	3.43	100
No	6.59	5.14	32
Total	4.67	4.04	132
Sexually compulsive			
Yes	6.27	5.03	73
No	9.14	7.58	14
Total	6.74	5.57	87
Total			
Yes	3.63	3.85	350
No	4.16	4.96	114
Total	3.76	4.15	464
*DASS21 Stress Scale*			
Non-compulsive			
Yes	5.64	4.04	177
No	5.66	4.36	68
Total	5.64	4.12	245
Moderately compulsive			
Yes	7.67	4.06	100
No	10.97	4.97	32
Total	8.47	4.51	132
Sexually compulsive			
Yes	11.81	4.95	73
No	12.71	6.40	14
Total	11.95	5.18	87
Total			
Yes	7.51	4.86	350
No	8.02	5.59	114
Total	7.63	5.05	464
*Religious and Spiritual Struggles Scale (RSSS)*			
Non-compulsive			
Yes	51.37	14.76	177
No	40.24	11.81	68
Total	48.28	14.84	245
Moderately compulsive			
Yes	72.50	11.91	100
No	65.03	15.66	32
Total	70.69	13.25	132
Sexually compulsive			
Yes	90.95	16.14	73
No	88.14	22.83	14
Total	90.49	17.26	87
Total			
Yes	65.66	21.32	350
No	53.08	22.40	114
Total	62.57	22.24	464

Mean and standard deviation for time spent viewing porn, DASS21 Scale Scores, Religious and Spiritual Struggle Scale (RSSS) Scores by sexual compulsivity categories for the religious and non-religious are presented in Table 2. Scores for the DASS-21 scales and RSSS are presented as sums of the items on the scale. Higher scores indicate higher degrees of Depression, Anxiety, Stress and Religious/Spiritual Struggle, respectively.

One-way between-subjects multivariate analysis of variance (MANOVA) was performed on the six dependent variables (age, time viewing porn, DASS-21 depression, DASS-21 anxiety, DASS-21 stress and RSSS) separately for the SCS groups and religious/non-religious groups. The independent variables were the religious/non-religious groups and three levels of sexual compulsivity: non-sexually compulsive (NSC), moderately sexually compulsive (MSC) and sexually compulsive (SC). Univariate results are presented in Table 3 for the following groups of dependent variables: age, religious attendance frequency, time viewing porn, DASS-21 depression, DASS-21 anxiety, DASS-21 stress and the RSSS scores.

**Table 3:** Multivariate and univariate analyses of variance and effect sizes for age, time spent viewing porn, DASS21 Scale Scores, Religious and Spiritual Struggle Scale Score by sexual compulsivity categories for the religious and non-religious

Source	Multivariate	Univariate					
		Age	Time spent viewing porn	DASS21 depression	DASS21 anxiety	DASS21 stress	Religious and Spiritual Struggles Scale
*Religious*							
Sexual compulsivity (*F*)	31.30**	2.88	11.06**	68.78**	33.94**	54.63**	213.43**
Size of effect (*η*_p_2)	0.35	0.02	0.60	0.28	0.16	0.24	0.55
*Non-Religious*							
Sexual compulsivity (*F*)	11.40**	1.49	1.73	15.94**	24.74**	29.85**	77.22**
Size of effect (*η*_p_2)	0.39	0.03	0.03	0.22	0.31	0.27	0.58

*p < 0.05; **p < 0.001

Although MANOVA is robust to unequal sample sizes, for the present study the sample sizes are so different for the religious (*n* = 350) and the non-religious (*n* = 114) it was determined that separate MANOVAs would be conducted for each religious group. Evaluation of the assumptions of normality, linearity, homogeneity of variance, covariance matrices and multi-collinearity was met satisfactorily. Effect sizes are reported in partial eta-squared terms. When multiplied by 100, these partial eta-squared coefficients may be interpreted as the percentage of variance of the effect accounted for by the dependent variable(s).

## Results

### Sexual Compulsivity

For the religious, the combination of dependent variables resulted in a statistically significant effect for the sexual compulsivity group (Wilks *λ* = 0.42, *F* (12, 684) = 31.30, *p* < 0.001) and accounted for a large association between the sexual compulsivity group and the dependent variables (*η*_p_2 = 0.35). For the non-religious, the combination of dependent variables also resulted in a statistically significant effect for the sexual compulsivity group (Wilks *λ* = 0.37, *F* (12, 212) = 11.40, *p* < 0.001) and accounted for a large association between the sexual compulsivity group and the dependent variables (*η*_p_2 = 0.39). Univariate Fs and effect sizes (*η*_p_2) for all independent variables by religious and non-religious groupings are reported in Table 3. There was a statistically significant relationship (with large effect) between sexual compulsivity and DASS-21 Depression, Anxiety, Stress and RSSS for both the religious and non-religious.

### Age

The mean age for the religious was 31.9 (SD = 15.7, range = 18 to 69 years of age); and for the non-religious 30.6 (SD = 12.7, range = 18 to 69 years of age). However, the differences in age between the religious and non-religious were not significant. For the religious, there was no significant difference in age between SCs, MSCs and NCs. Likewise, for the non-religious, there was no significant difference in age between SCs, MSCs and NCs.

### Time Spent Viewing IP

Between-subject effects are presented in Table 4. The non-religious (*M* = 11.86; SD = 11.79) spent significantly (*F* (1, 463) = 8.20, *p* = 0.004) more time viewing porn than the religious (*M* = 8.60; SD = 10.05). Though, the effect size was small, *η*_p_2 = 0.02 (see Table 4).

**Table 4:** Between-subject effects for religion and sexual compulsivity

Source	Type III sum of squares	df	Mean square	F	Sig	Partial eta squared
*Religion (yes/no)*						
Age	16.47	1.00	16.47	3.82	0.05	0.01
Hours viewing Internet pornography per week	866.55	1.00	866.55	8.20	0.00**	0.02
DASS21 Depression Scale	82.77	1.00	82.77	3.54	0.06	0.01
DASS21 Anxiety Scale	176.85	1.00	176.85	12.97	0.00**	0.03
DASS21 Stress Scale	121.84	1.00	121.84	6.32	0.01*	0.01
Religious and Spiritual Struggles Scale	3123.58	1.00	3123.58	15.09	0.00**	0.03
*SCS categories (religious)*						
Age	27.49	2.00	13.74	2.88	0.06	0.02
Hours viewing Internet pornography per week	2111.56	2.00	1055.78	11.06	0.00**	0.06
DASS21 Depression Scale	2986.50	2.00	1493.25	68.76	0.00**	0.28
DASS21 Anxiety Scale	844.83	2.00	422.42	33.94	0.00**	0.16
DASS21 Stress Scale	1971.20	2.00	985.60	54.63	0.00**	0.24
Religious and Spiritual Struggles Scale	87,480.05	2.00	43,740.02	213.43	0.00**	0.55
*SCS categories (Non-Religious)*						
Age	8.58	2.00	4.29	1.49	0.23	0.03
Hours viewing Internet pornography per week	475.65	2.00	237.83	1.73	0.18	0.03
DASS21 Depression Scale	914.68	2.00	457.34	15.94	0.00**	0.22
DASS21 Anxiety Scale	858.74	2.00	429.37	24.74	0.00**	0.31
DASS21 Stress Scale	964.92	2.00	482.46	20.85	0.00**	0.27
Religious and Spiritual Struggles Scale	33,001.37	2.00	16,500.69	77.22	0.00**	0.58

^*^p < 0.05; **p < 0.001

The religious SCs (12.96 h/week) spend significantly (*F* (2, 349) = 11.06, *p* < 0.001) more time viewing porn than both MSCs (8.98 h/week) and NCs (6.60 h/week). The effect size was medium, *η*_p_2 = 0.06. By contrast, the non-religious, (*F* (2, 113) = 1.73, *p* = 0.182, *η*_p_2 = 0.03), did not show any significant differences in time spent viewing porn between the sexual compulsivity groups (see Table 4).

The two groupings that viewed porn the most were the non-religious SCs (*M* = 14.59; SD = 20.62) followed by religious SCs (*M* = 12.96; SD = 14.59; see Table 2).

### DASS-21 Scores

The religious and non-religious depression scores did not show any significant differences. There was a statistically significant difference on the anxiety scores between the religious (*M* = 3.63; SD = 3.85) and non-religious (*M* = 4.16; SD = 4.96), *F* (1, 458) = 12.97, *p* < 0.001. The effect size was large; η_p_2 = 0.28. There was also a statistically significant difference on the stress scores based between the religious (*M* = 7.51; SD = 4.86), and non-religious (*M* = *M* = 8.02; SD = 5.59), *F* (1, 458) = 6.32, *p* < 0.05, *η*_p_2 = 0.014. The effect size (*η*_p_2 = 0.14) was also large (see Table 4).

For both the religious and non-religious, all DASS-21 scores were statistically significantly higher for SCs than for NCs. The depression, anxiety and stress score each rose as sexual compulsivity increased (Table 2). All effect sizes were large as presented in Table 4.

### Religious and Spiritual Struggles Scores (RSSS)

There was a statistically significant difference (*F* (1, 458) = 15.09, *p* < 0.001) on the RSSS scores between the religious (*M* = 65.66; SD = 21.31) and non-religious (*M* = 53.08; SD = 22.40). The effect size was small; *η*_p_2 = 0.03 (see Table 4).

For both the religious and non-religious, RSSS scores were statistically significantly higher for SCs than for NCs. The RSSS scores rose as sexual compulsivity increased (Table 2). All effect sizes were large as presented in Table 4.

### Religious Affiliation and Religious Attendance Frequency

In the demographic questionnaire, participants were asked about their religious preference (if any) and religious attendance frequency. One-way between-subjects analysis of variance (ANOVA) was performed on the dependent variables of religious preference (if any) and religious attendance frequency with the independent variable of sexual compulsivity.

#### Religious Affiliation by Sexual Compulsivity

Sexual compulsivity among various religious affiliations is shown in Table 5. The mean difference was significant at the 0.05 level between “None/Declined to State” and “Islamic.” However, this result is limited by the small sample size (*n* = 17) for the Islamic group. For the groups “None/Declined to State” (*n* = 95) and “Other” (*n* = 63), the mean difference was significant at the 0.05 level. In this case, the sample size was large enough to warrant a valid and reliable result. There were no statistically significant differences between the other groups for the dependent variable sexual compulsivity (see Table 5).

**Table 5:** Religious affiliation by sexual compulsivity categories

	Non-sexually compulsive (%) (*n* = 245)	Moderately sexually compulsive (%) (*n* = 132)	Sexually compulsive (%) (*n* = 87)
*Religious affiliation*			
Protestant (*n* = 125)	52.0	28.8	19.2
None/Declined To State^a^ (*n* = 95)	63.2	27.4	9.5
Catholic (*n* = 65)	63.1	21.5	15.4
Other^a^ (*n* = 63)	38.1	36.5	25.4
Mormon (*n* = 47)	42.6	38.3	19.1
Christian Scientist (*n* = 24)	62.5	16.7	20.8
Muslim (*n* = 17)	23.5	29.4	47.1
Hindu (*n* = 11)	63.6	18.2	18.2
Jewish (*n* = 9)	55.6	33.3	11.1
Buddhist (*n* = 8)	50.0	12.5	37.5

^a^The mean difference was significant at the 0.05 level between “None/Declined to State” and “Islamic.” Likewise between “None/Declined to State” and “Other,” the mean difference was significant at the 0.05 level

#### Religious Attendance Frequency by Sexual Compulsivity

There were no statistically significant differences between the religious attendance frequencies for the dependent variable sexual compulsivity.

## Discussion

This study sought to examine the sexual compulsivity and emotional, spiritual distress of religious and non-religious adults who sought assessment for pornography addiction on the Internet. The data for the present study included sufficiently large sample sizes. In this situation, it is important to examine not only statistical significance, but also size of effects to determine the meaningfulness of findings. The following discussion of findings emphasizes the interpretation of size of effect.

### Age

The religious and non-religious males and females in this study did not differ significantly in age. Thus, age was not a very meaningful variable. This is consistent with previous findings (Cooper et al. 2000) that male and female SCs and NCs that evidence some interest in IP use tend to be in their early 30 s with an age range of between early 20 s to early 40 s.

### Sexual Compulsivity and Time Spent Viewing IP Use

The non-religious spent significantly more time viewing porn than the religious. This result agreed with the findings of Short et al. (2015) and Nelson et al. (2010) who found that religious individuals were less likely to have ever used or to currently be using porn. Abell et al. (2006) found heavy IP use (6 h or more) occurring among conservative Christian males (3.4%). In their study, these were about half of the general male Internet using population (6.5%; Cooper et al. 2002).

**Fig. 1:**
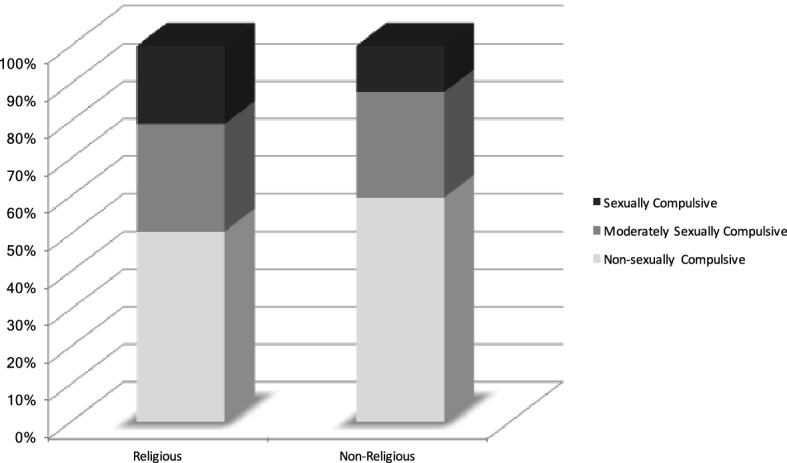
Sexual compulsive categories by percentage for the religious and non-religious

Yet, the religious in the present study were more sexually compulsive (Fig. 1). The percentage of sexual compulsivity users for the non-religious (12.30%) was significantly lower than the religious (20.80%). This finding squared with a study of religious cyberporn users by Abell et al. (2006). They discovered a statistically significant positive relationship between spiritual beliefs scores and sexual compulsivity (also measured using Kalichman’s SCS) of cyberporn users who identified as evangelical Christians.

One possible explanation for the higher compulsivity among the religious is that with their stronger spiritual beliefs, they are more likely to engage in solitary cyberporn activity because they view such behavior as permissible or at least less objectionable than extramarital sexual activity with a partner. They become more attached to the compulsive experience of viewing porn. It may be that the online sexual outlet limits cognitive dissonance that would be expected among those with stronger spiritual beliefs (Abell et al. 2006).

For the religious, the combination of dependent variables resulted in a statistically significant effect for the sexual compulsivity group (Wilks *λ* = 0.52, *F* (22, 674) = 12.06, *p* < 0.001) and accounted for a large association between the sexual compulsivity group and the dependent variables (*η*_p_2 = 0.35). For the non-religious, the combination of dependent variables resulted in a statistically significant effect for sexual compulsivity group (Wilks *λ* = 0.31, *F* (22, 202) = 7.37, *p* < 0.001) and accounted for a large association between the sexual compulsivity group and the dependent variables (*η*_p_2 = 0.39).

All of the dependent variables, except age, were significantly higher for SCs than NCs for the religious. For the non-religious, all of the dependent variables, except age and time spent, were significantly higher for SCs than NCs.

### Emotional and Spiritual Distress

**Fig. 2:**
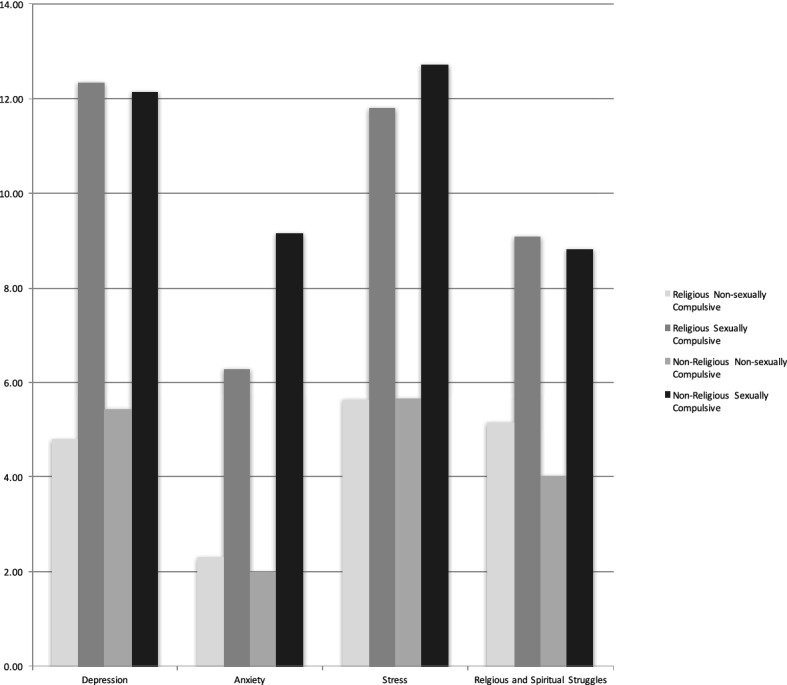
Dependent variables by religious status and sexual compulsive category. All measures were transformed to the same scale to allow for comparisons

Comparison of the DASS-21 and RSS results for the 2 × 2 categories of religious/non-religious and non-compulsive/sexually compulsivity is graphically represented in Fig. 2. All measures were transformed to the same scale to allow for comparisons. The religious and non-religious depression scores did not show any significant differences. Though, the non-religious were significantly more anxious and stressed than between the religious (Fig. 2). Perhaps, religious beliefs and/or rituals provide some means for discharging anxiety and relieving stress.

In striking contrast, sexual compulsivity accounted for the largest amount of variance for the religious (*η*_p_2 = 0.35) and non-religious (*η*_p_2 = 0.39). As the online sexual compulsivity increased, there was an increase in depression, anxiety, stress and religious/spiritual struggles. These results show that consequences and emotional fallout from online sexual compulsivity are universally felt regardless of religiosity.

Emotional and/or spiritual distress can lead to the development of CIPU as means of coping with life. In the present study, compulsive IP users who were more emotionally and/or spiritually distressed may have chosen IP use to replace or avoid real sexual relationships. Carnes (2001) found that cybersex users may actually prefer virtual sex to avoid the complications of a real relationship. A sexual relationship requires emotional vulnerability and tolerance of stress. IP use, on the other hand, is a come-as-you-are experience. Thus, the appeal of IP use may be particularly strong for a person who is unwilling or unable to engage emotionally and bodily in a relational sexual experience. Carnes (2001) found the cybersex disrupts the intimacy building and courtship process of sexual relating. Also, as Reid and Woolley (2006) noted, “several aspects of the sexual experience require risk taking and vulnerability” (p. 219).

Conversely, over time CIPU may lead to or causing emotional and/spiritual distress. Evidence from numerous case examples (Carnes 2001; Laaser and Gregoire 2003) supports the conclusion that compulsive sexual behaviors lead to depression. This finding parallels those of other studies that the consequences of compulsive sexual behaviors are far reaching and result in compromised relationships, family breakups, work difficulties and financial troubles, legal problems, loss of interest in things not sexual, low self-esteem and despair (Adams and Robinson 2001; Carnes 1991; Reid and Woolley 2006).

Even if CIPU does not directly cause depression, it may indirectly make it more likely as fallout from the consequences. Excessive IP use impacts all domains of life: personal, relational and professional (Manning 2006; Twohig et al. 2009). It is clear that these negative consequences could contribute to more emotional and spiritual distress.

### Religious and Spiritual Struggle and Distress

There was a statistically significant difference on the RSSS scores between the religious and non-religious as illustrated in Fig. 2. The effect size was large, and percentage difference is visually noticeable. At each level of compulsivity, the religious appear to have more religious and spiritual struggle than the non-religion. One likely cause of this increased struggle and distress is that IP use is out of step with the values of the religious person and internal conflict arises.

Religion can either create positive or negative dynamics concerning mental health issues (Abraham 1994; Birchard 2004; Templin and Martin 1999). Abraham (1994) contends that the aspect of religiosity can not only have the capability to enhance mental health for the individual as related to sexuality; but likewise, can also disconnect and isolate the person from her/his sexuality, which can lead to various problems.

Religious persons in this study may come to see that their sexuality is wrong and sinful. The dynamics such as guilt and shame integrate into one’s religious beliefs and can also influence sexual behaviors in a detrimental manner. Birchard (2004) discusses how for the religious individual there may be compulsive cycles in which there are control and release aspects in sexual behavior, which can exacerbate these problems. He argues that sexual addiction and religious behavior are interlinked in that the sexual behavior represents the release part of the cycle and the religious behavior represents the control part of the cycle. “The characteristics of the religious behavior (i.e., repentance, confession, diligence, service) actually move the client to a sense of neediness or entitlement and thus back into the sexually addictive behavior. Unless this cycle is understood and aborted these apparently antithetical behaviors contribute to one another” (p. 86). Thus, external judgment or internalized guilt and shame from religious ideology may have pushed religious individuals in this study further into their addictive behavior. Twelve step groups may help these individual share guilt and shame feelings in a non-judgmental environment.

### Limitations

The instruments administered relied on self-report by subjects, which is probably the largest limitation of this study. We have no way of knowing whether the data collected is an accurate representation of the online community and therefore cannot make sweeping generalizations about sexuality and the Internet. In addition, while online methodologies for research continue to improve, it is impossible to know who has filled out the survey, whether they were being honest, and whether they filled out the survey on multiple occasions. Data cleaning was performed to minimize the effects of this limitation, however, it still existed. With no ability to see the subjects or follow-up their responses, it may be difficult to recognize if other contaminating variables may have influenced the way the instrument was completed. For example, an individual may have been in crisis when completing the survey, or was significantly depressed or anxious.

## Conclusion

In conclusion, the non-religious spent significantly more time viewing IP than the religious. This finding corroborated with past research. Yet, the religious were significantly more sexually compulsive. This finding was both supported in previous research and other studies show no difference in compulsivity. Specific religious affiliations did not have any significant bearing on the degree of sexual compulsivity. The sexual compulsivity between individuals of various religious affiliations (Catholics, Protestants, Mormons, Christian Scientists, Muslims, Hindus, Jews and Buddhists) did not vary significantly; varied only the between unaffiliated and “Other” religious category. Online sexual compulsivity was associated with greater emotional and spiritual distress regardless of religiosity. The degree of emotional and spiritual distressed increased as sexual compulsivity increased. The religious and non-religious depression scores did not show any significant differences. Though, the non-religious were significantly more anxious and stressed than the between the religious. Religious practice is associated with less viewing of IP, suggesting the likelihood that moral reasons may provide some rationale for not viewing IP. At the same time, religious practice might reinforce shame in the addiction cycle thus religious individuals may be more at-risk to developing a compulsive pattern of viewing IP. Lastly, we suggest future research should investigate the degree to which shame may be higher in religious individuals who have problematic IP use. Also how unconditional acceptance and other shame-reducing practices of 12-step recovery groups may reduce shame and in turn reduce compulsive IP use.
